# Leaf Soluble Carbohydrates, Free Amino Acids, Starch, Total Phenolics, Carbon and Nitrogen Stoichiometry of 24 Aquatic Macrophyte Species Along Climate Gradients in China

**DOI:** 10.3389/fpls.2019.00442

**Published:** 2019-04-11

**Authors:** Qingchuan Chou, Te Cao, Leyi Ni, Ping Xie, Erik Jeppesen

**Affiliations:** ^1^Donghu Experimental Station of Lake Ecosystem, State Key Laboratory of Freshwater Ecology and Biotechnology, Institute of Hydrobiology, Chinese Academy of Sciences, Wuhan, China; ^2^College of Life Sciences, University of Chinese Academy of Sciences, Beijing, China; ^3^Department of Bioscience, Aarhus University, Silkeborg, Denmark; ^4^Sino-Danish Center for Education and Research, University of Chinese Academy of Sciences, Beijing, China

**Keywords:** aquatic macrophytes, life form, stoichiometry, biogeographical, climate gradients

## Abstract

Leaf soluble carbohydrates (SC), free amino acids (FAA), starch, total phenolics (TOPH), carbon (C), and nitrogen (N) stoichiometry of 24 aquatic macrophyte species were studied at 52 selected sites in eastern, 31 sites in southwestern and 6 sites in western China, including 12 submerged, 6 floating-leaved, 4 emergent and 2 free-floating macrophytes. The leaf stoichiometric characteristics differed significantly among the plant species of the four different life forms, the lowest C content occurring in submerged macrophytes and the highest N content in free-floating macrophytes. Overall, though the variance explained by the linear regression models was low, the C and N contents decreased toward the northern latitudes, the C content and the C:N ratios increased with increasing altitude. Multiple regressions revealed that the stoichiometric characteristics of submerged macrophytes varied significantly across the large spatial and climatic gradients and among the species studied. For floating-leaved and emergent macrophytes, no correlation between climate factors and SC, FAA, starch, TOPH, C, and N contents and C:N ratio was observed. For free-floating macrophytes, the TOPH content was markedly positively correlated with latitude and altitude. We conclude that the C and N contents related more closely to latitude, altitude or mean annual air temperature than did the C and N metabolic indicators for the submerged macrophytes, while the relationships with the metabolic indicators turned out to be insignificant for most species of the other life forms. The results helped us to identify species with significant physiological plasticity across geographic and climatic gradients in China, and such information is useful when conducting restoration of lost aquatic plants in different climate regions.

## Introduction

Generally, plant nutrient status has been evaluated by examining nutrient contents in plant tissues (Duarte, 1992; Gerloff and Krombholz, 1966). Ecological stoichiometry provides an integrative approach to explore the relationships between plants and their environment using parameters such as species composition and distribution, population dynamics, food web and biogeochemistry at various spatial scales (Sterner et al., 1992; Elser et al., 2000, 2010; Sterner and Elser, 2002). In freshwater and terrestrial ecosystems, the contents of mineral elements in organisms have been frequently studied (Jackson et al., 1991; Duarte, 1992; Mcjannet et al., 1995; Aerts and Chapin, 1999), particularly the major elements such as carbon (C), nitrogen (N), and phosphorous (P), which are the key nutrients supporting the life of organisms and vital to the ecological functions of ecosystems (Reich and Oleksyn, 2004; Han et al., 2005, 2011; He et al., 2006). Temperature is an important factor affecting the biological activity and nutrient metabolism of both individual organisms and ecosystems, and stoichiometric patterns in plant tissues have been observed to differ across large geographical and climatic scales, reflecting variation in biological activity and the biogeochemical cycling of essential elements such as N and P (Sterner and Elser, 2002; Reich and Oleksyn, 2004; Han et al., 2005, 2011).

Macrophytes are fundamental components affecting food webs and functions in many shallow aquatic ecosystems (Jeppesen et al., 1998; Frost and Hicks, 2012). Spatial heterogeneity in the sediment N content caused by eutrophication may affect the metabolic activity of the plants as well as intermediate metabolites such as soluble carbohydrate (SC), starch, free amino acids (FAA), phenolic compounds (TOPH) and the N-related stoichiometries (Sterner and Elser, 2002; Cronin and Lodge, 2003; Cao et al., 2008; Li et al., 2015). SC and starch serve as a storage of energy, as C reservoir and as structural components (Coolidge-Stolz et al., 2002). Carbohydrates provide carbon skeleton and energy for the synthesis of amino acids that play a central role in the metabolism of C and N by acting as a nitrogen transporter and reservoir and as precursors for proteins and many secondary metabolites (Cao et al., 2008). The FAA content in aquatic plants can be affected by the balance between light and N availability and generally increases when plants are exposed to environmental stressors causing reduced growth (Lea and Forde, 1994; Lam et al., 1998; Foyer et al., 2003). The FAA content is therefore used as a physiological indicator in plants. Phenolic compounds are important secondary metabolites of plants and act as precursors for lignin synthesis and antioxidants (Hong et al., 2016; Adbelrahman et al., 2017). High light and carbon dioxide and low nitrogen availabilities, as well as plant damage caused by herbivorous fish, generally increase the content of phenolic compounds in plant tissues (Dudt and Shure, 1994; Cronin and Lodge, 2003). Thus, in addition to elementary stoichiometry, examination of the contents of SC, FAA, starch, and TOPH may help gain insight into the C and N metabolic strength of macrophytes.

Compared with the great attention paid to terrestrial plants few studies have focused on the stoichiometric characteristics of aquatic macrophytes that occur over large geographic scales (Elser et al., 2000; Reich and Oleksyn, 2004; Han et al., 2005, 2011; He et al., 2006) and thus are exposed to significant differences in climate and human activities (Proctor, 1982; Wu et al., 1999; Lacoul and Freedman, 2006; Frost and Hicks, 2012; Sardans et al., 2012). However, some experiments and field investigations on aquatic plants have attempted to define the range and variation of their physiological state by exploring various metabolic indicators. The emphasis of these studies has been placed on the stoichiometry differences between various species (Fernández-Aláez et al., 1999; Li et al., 2015), the relationship between the contents of various elements (Frost and Hicks, 2012; Li et al., 2014; Xia et al., 2014) and the influence of lake sediment and water column nutrient gradients on plant stoichiometry (Qiu et al., 2013; Xing et al., 2013; Su et al., 2016). So far, though, no studies have dealt with the differences in stoichiometry of different life forms of aquatic plants over large geographic and climatic gradients. In this investigation, we examined the contents of SC, FAA, starch, TOPH, C, and N in the leaves of 24 aquatic macrophytes across large geographic and climatic gradients in China with the aim to elucidate plant C and N stoichiometry. We hypothesized that (1) plant biochemical and stoichiometric parameters would vary noticeably across latitude and altitude and with mean annual air temperature (MAT), and that (2) latitude, altitude and MAT would affect the C to N stoichiometry more consistently in submerged than in emergent, floating-leaved and free-floating macrophytes due to the fact that submerged macrophytes live below the water surface where temperature is less variable than at the water-air interface and above, and water availability is not of importance.

## Materials and Methods

### Study Area

**FIGURE 1:**
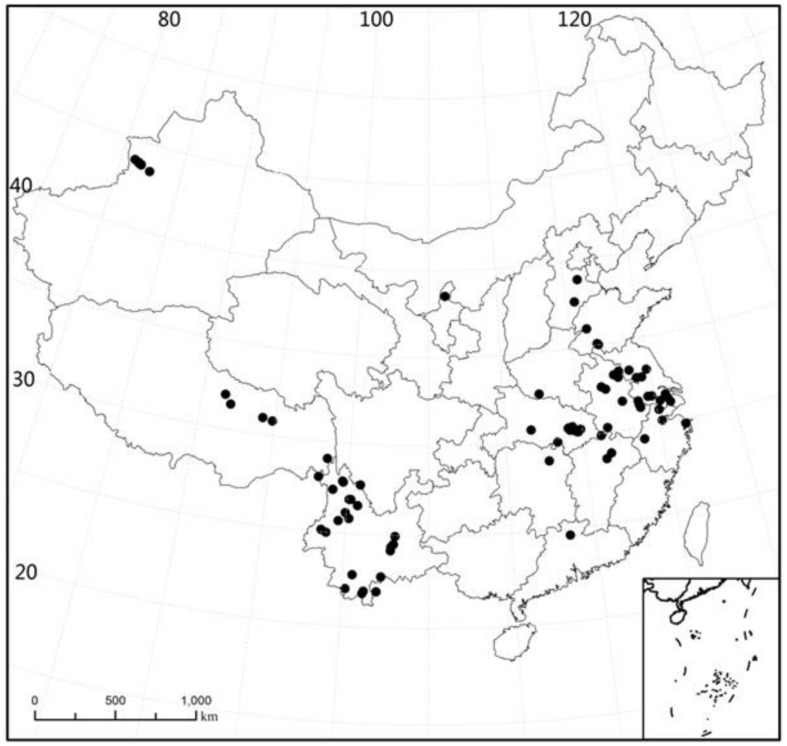
Sampling location of all study sites. Provincial boundaries are shown.

The plant samples were collected at 89 sites in China (21°26′ – 43°57′N, 80°39′−121°39′E) during the period 2003 to 2009. The sites were located in 15 major provinces, with 26 sites in Yunnan, 1 site in Guangdong, 1 site in Hunan, 2 sites in Jiangxi, 4 sites in Zhejiang, 12 sites in Hubei, 1 site in Henan, 2 sites in Hebei, 6 sites in Shandong, 9 sites in Anhui, 11 sites in Jiangsu, 6 sites in Xinjiang, 5 sites in Tibet, 1 site in Ningxia, and 1 site in Shanghai (Figure 1). For all sampling sites, the MAT range was −3 to 22.4°C (mean: 14.8°C, median: 15.5°C) and the span of altitude was 1.0 to 4477 m (mean: 695.7 m, median: 25 m).

### Aquatic Plant Sampling and Biochemical Analysis

We collected all species present at the sites during the same season (summer) of the year to avoid the impact of inter-seasonal differences in climate. At each sampling site, latitude and altitude were recorded using a portable GPS (Garmin 60csx), and MAT was derived from the website of the meteorological department^1^. Unfortunately we have no nutient data for lakes as the plants used were sampled with the purpose of describing species distribution.

We took macrophyte samples using a reaping hook in quadrat areas sized 0.2 m^2^. Three replicates were taken at each site and sorted into species. Not all sites hosted all species. The aquatic macrophytes sampled in lakes and rivers included four life forms: 12 submerged macrophytes (*Potamogeton pectinatus*, *P. perfoliatus*, *P. lucens*, *P. malaianus*, *P. maackianus*, *P. crispus*, *Najas minor*, *Najas marina*, *Hydrilla verticillata*, *Ceratophyllum demersum*, *Myriophyllum verticillatum*, and *Vallisneria natans*), 6 floating-leaved macrophytes (*Potamogeton natans*, *Trapa bispinosa*, *Euryale ferox*, *Hydrocharis dubia*, *Nymphoides peltatum*, and *Polygonum amphibium*), 4 emergent macrophytes (*Zizania latifolia*, *Nelumbo nucifera*, *Scirpus validus*, and *Typha orientalis*) and 2 free-floating macrophytes (*Eichhornia crassipes* and *Lemna minor*). The macrophytes were collected, sorted into species, washed gently and brought to the laboratory where they were oven-dried at 80°C for 72 h to constant weight for further analysis (Su et al., 2016). A total of 392 samples were taken– 241, 90, 16, and 45 samples of submerged, floating-leaved, free-floating and emergent macrophytes, respectively. The dry samples of leaves were grounded into fine powder using a pestle and mortar for the analysis of SC, FAA, starch, TOPH, C, and N. Of each powder, about 100 mg was extracted with 10 mL 80% ethanol at 80°C for 20 min and then centrifuged for 15 min at 5,000 g (Cao et al., 2008, 2009). After centrifugation, the supernatant was used for determination of SC and FAA contents after reacting with anthrone and ninhydnn, respectively (Yemm and Willis, 1954; Yemm and Cocking, 1955) using alanine and glucose as standards. The supernatant was used for measurement of TOPH following the method described by Mole and Waterman (1987). Tannic acid (Sigma Chemical Company) was used as standard. The residue was used for the analysis of starch content following the method of Dirk et al. (1999). The C and N contents of all samples were determined applying an elemental analyzer (Flash EA 1112 series, CE Instruments, Italy).

### Data Analysis

We compared the statistical differences in leaf SC, FAA, starch, TOPH, C, and N and C:N (mass:mass) ratio for all the species and when divided into the four different life forms. To characterize the biogeographical gradient patterns of leaf stoichiometry, we first log-transformed (base *e*) the data of latitude, altitude, MAT [ln(MAT+5)], leaf SC, FAA, starch, TOPH, C, and N content and the C:N ratio for each species at each sampling site and then performed linear regressions for all the species and stepwise regressions for all the species, different life forms and specific species. Bonferroni correction was made as we tested three independent hypotheses on the same set of data. We also explored distribution patterns in the plant characteristica mentioned above.

The analyses were conducted with IBM SPSS Statistics 22. A sampling site map was produced using ArcMap 10.2. All figures were plotted using OriginPro 9.0.

## Results

### The Contents of SC, FAA, Starch, TOPH, C, and N and the C:N Ratios of Aquatic Plants Across All Species

**FIGURE 2:**
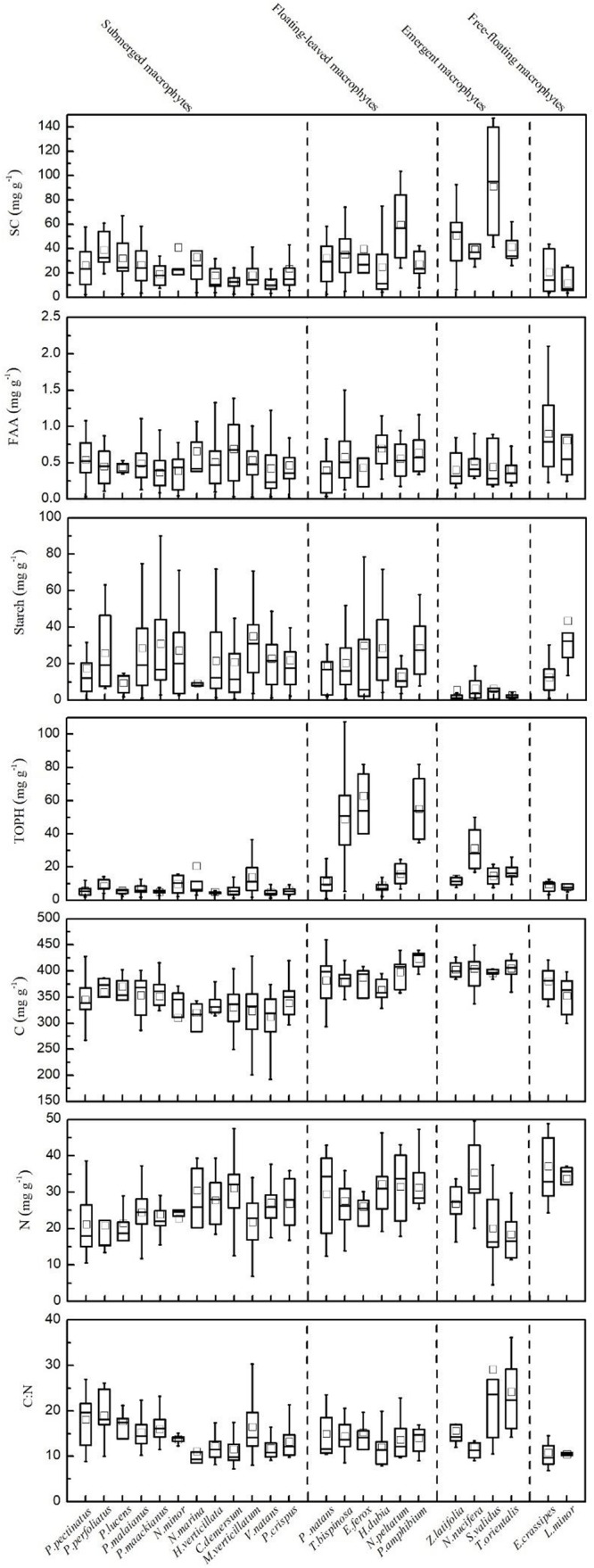
Leaf stoichiometric characteristics for all 24 aquatic plant species. Leaf soluble carbohydrates (mg g^–1^), free amino acids (mg g^–1^), starch (mg g^–1^), total phenolics (mg g^–1^), carbon (mg g^–1^), nitrogen (mg g^–1^) and the C:N ratio, mean ± SD.

The contents of SC, FAA, starch, TOPH, C, and N and the C:N ratio in the leaves of the aquatic macrophytes varied greatly (Figure 2, Supplementary Figure S1, and Supplementary Table S1). The contents of SC, FAA, TOPH, C, and N and the C:N ratios fitted log-normal distributions, while an exponential model was most reliable for starch (Supplementary Figure S1). The coefficients of variation (CV) for the contents of SC, FAA, starch, TOPH, C, and N and the C:N ratios were 0.83, 0.64, 1.05, 1.17, 0.14, 0.32, and 0.44, respectively (Supplementary Figure S1 and Supplementary Table S2).

**FIGURE 3:**
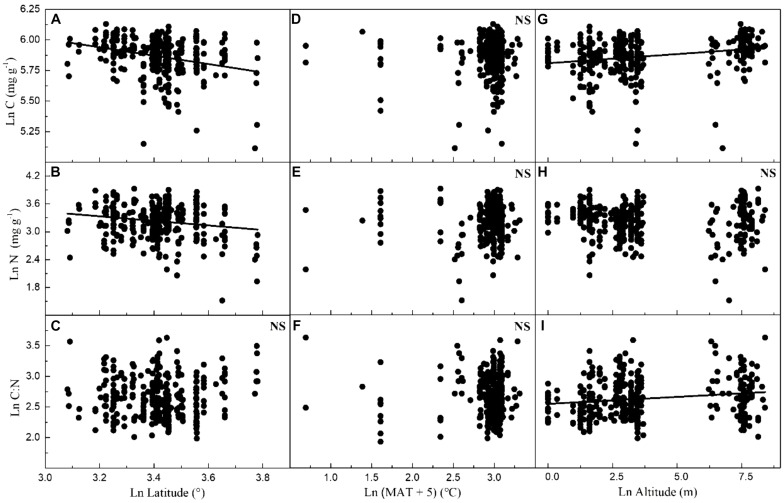
Relationships between leaf carbon and nitrogen contents and the C:N ratio of plants, latitude, mean annual temperature and altitude in China. Each data point represents a value of all observations of carbon and nitrogen at each sampling site (Figure 1). Linear regressions are shown for **(A)** latitude and leaf carbon (*r*^2^ = 0.080, *n* = 339, *P* < 0.001); **(B)** latitude and leaf nitrogen (*r*^2^ = 0.027, *n* = 339, *P* = 0.001); **(C)** latitude and C:N ratio (*r*^2^ = –0.002, *n* = 338, *P* = 0.634); **(D)** MAT and leaf carbon (*r*^2^ = –0.002, *n* = 347, *P* = 0.669); **(E)** MAT and leaf nitrogen (*r*^2^ = –0.002, *n* = 347, *P* = 0.336); **(F)** MAT and C:N ratio (*r*^2^ = –0.002, *n* = 346, *P* = 0.595); **(G)** altitude and leaf carbon (*r*^2^ = 0.059, *n* = 339, *P* < 0.001); **(H)** altitude and leaf nitrogen (*r*^2^ = 0.003, *n* = 339, *P* = 0.172); **(I)** altitude and C:N ratio (*r*^2^ = 0.027, *n* = 338, *P* = 0.001). All dates were log-transformed (base *e*). Bonferroni correction significance level threshold value, *P* = 0.05/3.

**FIGURE 4:**
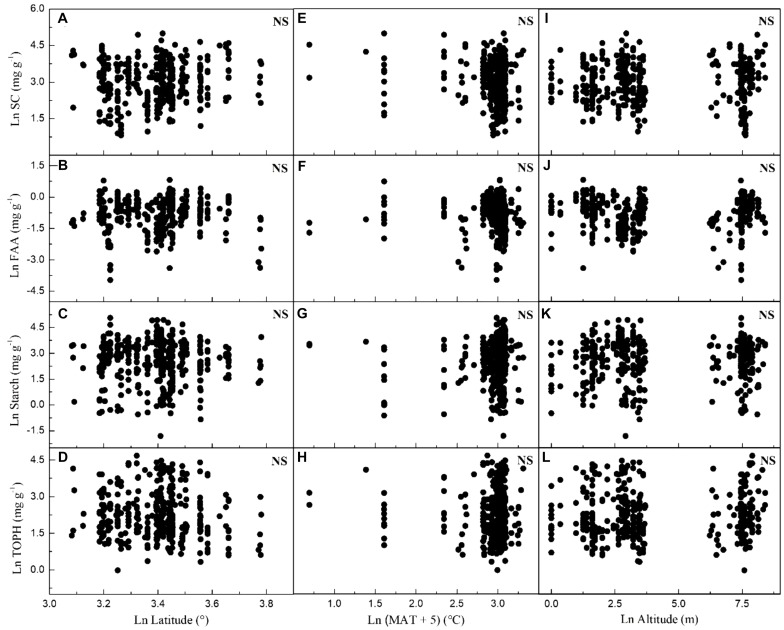
Relationships between leaf soluble carbohydrates, free amino acids, starch and total phenolics contents of plants, latitude, mean annual temperature and altitude in China. Each data point represents a value of all observations of soluble carbohydrates, free amino acids, starch and total phenolics at each sampling site (Figure 1). Linear regressions are shown for **(A)** latitude and leaf soluble carbohydrates (*r*^2^ = 0.008, *n* = 383, *P* = 0.043); **(B)** latitude and leaf free amino acids (*r*^2^ = –0.002, *n* = 371, *P* = 0.670); **(C)** latitude and leaf starch (*r*^2^ = –0.002, *n* = 376, *P* = 0.814); **(D)** latitude and leaf total phenolics (*r*^2^ = 0.005, *n* = 383, *P* = 0.098); **(E)** MAT and leaf soluble carbohydrates (*r*^2^ = 0.011, *n* = 392, *P* = 0.020); **(F)** MAT and leaf free amino acids (*r*^2^ = –0.002, *n* = 380, *P* = 0.784); **(G)** MAT and leaf starch (*r*^2^ = 0.009, *n* = 385, *P* = 0.031); **(H)** MAT and leaf total phenolics (*r*^2^ = –0.002, *n* = 392, *P* = 0.653); **(I)** altitude and leaf soluble carbohydrates (*r*^2^ = –0.003, *n* = 383, *P* = 0.828); **(J)** altitude and leaf free amino acids (*r*^2^ = 0.002, *n* = 371, *P* = 0.180); **(K)** altitude and leaf starch (*r*^2^ = –0.002, *n* = 376, *P* = 0.736); **(L)** altitude and leaf total phenolics (*r*^2^ = –0.002, *n* = 383, *P* = 0.298). All dates were log-transformed (base *e*). Bonferroni correction significance level threshold value, *P* = 0.05/3.

Considering all macrophytes together, latitude correlated negatively with the C and N contents (*p* < 0.05/3; Figure 3, Table 1, and Supplementary Table S3), whereas no such correlation was found for the SC, FAA, starch, and TOPH contents (Figure 4 and Table 1). Altitude correlated positively with the C content and the C:N ratio (*p* < 0.05/3 for both; Figure 4, Table 1, and Supplementary Table S3), but no correlation between altitude and N, SC, FAA, starch, and TOPH contents was observed (Figure 4 and Table 1). However, there was no significant relationship between MAT and the stoichiometric characteristics (Figures 3, 4 and Table 1).

**TABLE 1:** Linear regression models explaining the average leaf soluble carbohydrates, free amino acids, starch, total phenolics, carbon and nitrogen contents and the C:N ratio of all species included in the study.

Dependent variable	*df*	*P*	*r*^2^_adj_	Linear model
Ln SC	(1, 381)	0.002	0.023	4.599*** – 0.540** ln MAT+5
Ln C	(1, 337)	¡0.001	0.079	7.037*** – 0.343*** ln latitude
	(2, 336)	¡0.001	0.091	6.712*** – 0.257*** ln latitude + 0.008* ln altitude
Ln N	(1, 337)	0.001	0.027	4.912*** – 0.493** ln latitude
	(2, 336)	¡0.001	0.060	6.105*** – 0.808*** ln latitude – 0.031*** ln altitude
Ln C:N	(1, 336)	0.001	0.028	2.547***+ 0.023** ln altitude
	(2, 335)	¡0.001	0.044	1.083 + 0.034*** ln altitude + 0.417* ln latitude

The models result from a forward stepwise selection procedure using the independent variables latitude, altitude, and mean annual temperature. All significant (*P* < 0.05/3) regression models are shown. All dates were log-transformed (base *e*), the significance of the regression cofficients is indicated by *P < 0.05, **P < 0.01, ***P < 0.001, without asterisk *P* > 0.05. Bonferroni correction significance level threshold value, *P* = 0.05/3.

### The Contents of SC, FAA, Starch, TOPH, C, and N and the C:N Ratios of Aquatic Macrophytes With Different Life Forms and Species

**FIGURE 5:**
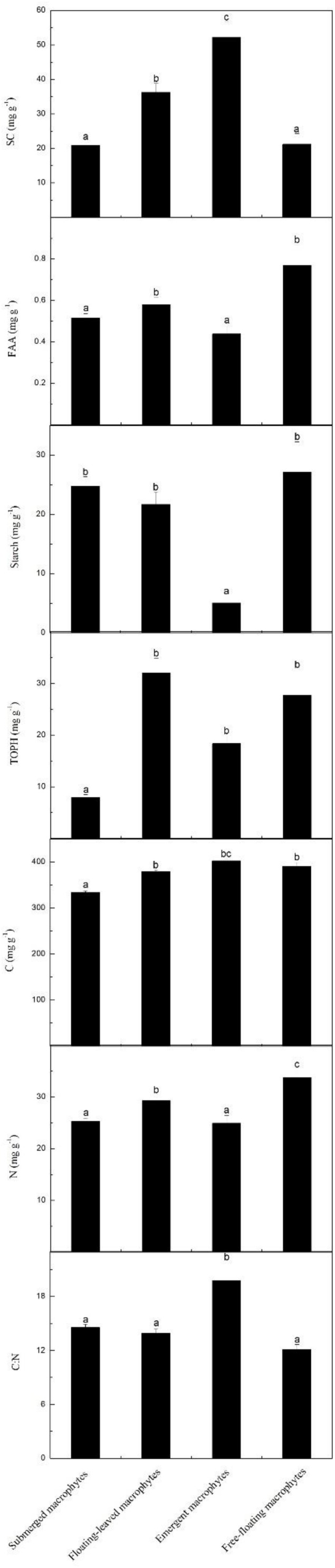
Leaf stoichiometric characteristics across four different macrophyte life forms. Leaf soluble carbohydrates (mg g^–1^), free amino acids (mg g^–1^), starch (mg g^–1^), total phenolics (mg g^–1^), carbon (mg g^–1^), nitrogen (mg g^–1^) and the C:N ratio, mean ± SE. Different letters indicate significant differences.

The contents of SC, FAA, starch, TOPH, C, and N and the C:N ratios differed significantly among the macrophytes with different life forms (Figures 2, 5). Among the four life forms, emergent macrophytes had the highest SC and the lowest starch contents (Figure 5). The FAA content of floating-leaved and free-floating macrophytes was significantly higher than that of submerged and emergent macrophytes (Figure 5). Submerged macrophytes had the lowest TOPH and C contents, free-floating macrophytes had the highest N content and emergent macrophytes the highest C:N ratio (Figure 5). Of all the species, *S. validus* had the highest SC content and the highest C:N ratio, *E. crassipes* had the highest FAA content, *T. orientalis* and *V. natans* had the lowest starch and TOPH contents, respectively, and *N. minor* and *L. minor* had the highest C and N contents, respectively (Figure 2).

Overall, multiple regressions revealed that the stoichiometric characteristic of the submerged macrophytes varied significantly across the large spatial and climatic gradients studied (Tables 2, 3). For submerged macrophytes, the stepwise regression revealed that the contents of SC and TOPH correlated negatively with MAT (*p* < 0.05/3; Table 2) and that the C, N, and TOPH contents had a marked negative correlation with latitude (*p* < 0.05/3 for both; Table 2 and Supplementary Table S4), while the C content and the C:N ratios correlated positively with altitude and the N content negatively with altitude (*p* < 0.01 for all; Table 2 and Supplementary Table S4). Significant linear trends between the C and N contents and the C:N ratios of three submerged species (*P. maackianus*, *P. crispus*, and *M. verticillatum*) and latitude, MAT and/or altitude were found (*p* < 0.05/3; Table 3). For floating-leaved and emergent macrophytes, no correlation between climate factors and SC, FAA, starch, TOPH, C, and N contents and C:N ratio was observed, but when refining to species level, the FAA, C, and N contents of *T. bispinosa*, *H. dubia* and *N. peltatum* demonstrated linear relationship with latitude, MAT and/or altitude (*p* < 0.05/3 for all; Table 3), the SC, TOPH and N contents and the C:N ratios of *T. orientalis* exhibited marked linear trends with altitude (*p* < 0.05/3 for both; Table 3). For free-floating macrophytes, the TOPH content had a marked positive correlation with latitude and altitude (*p* < 0.05/3 for both; Table 2), while the TOPH content and C:N ratio of *L. minor* increased with increasing latitude and MAT, respectively (*p* < 0.05/3 for both; Table 3).

**TABLE 2:** Linear regression models explaining the average leaf soluble carbohydrates, free amino acids, starch, total phenolics, carbon and nitrogen contents and the C:N ratio of different life forms.

Life form	Dependent variable	*df*	*P*	*r*^2^_adj_	Linear model
Submerged macrophytes	Ln SC	(1, 235)	0.013	0.022	4.349*** – 0.532* ln MAT+5
	Ln TOPH	(1, 235)	¡0.01	0.052	5.791*** - 1.172*** ln latitude
		(2, 234)	¡0.001	0.079	8.152*** – 1.390*** ln latitude – 0.546** ln MAT+5
	Ln C	(1, 201)	¡0.001	0.151	7.465*** – 0.486*** ln latitude
		(2, 100)	¡0.001	0.170	7.073*** – 0.383*** ln latitude + 0.011* ln altitude
	Ln N	(1, 201)	0.004	0.035	3.279*** – 0.028** ln altitude
		(2, 200)	¡0.001	0.089	6.018*** – 0.046*** ln altitude – 0.780*** ln Latitude
	Ln C:N	(1, 201)	¡0.001	0.110	2.448***+ 0.047*** ln altitude
Free-floating macrophytes	Ln TOPH	(1, 24)	0.009	0.224	−10.583*+ 4.076** ln latitude
		(2, 23)	0.004	0.332	−19.143**+ 6.302** ln latitude + 0.190* ln altitude

The models result from a forward stepwise selection procedure using the independent variables latitude, altitude and mean annual temperature. All significant (*P* < 0.05/3) regression models are shown. All dates were log-transformed (base *e*), the significance of the regression cofficients is indicated by *P < 0.05, **P < 0.01, ***P < 0.001, without asterisk *P* > 0.05. Bonferroni correction significance level threshold value, *P* = 0.05/3.

**TABLE 3:** Linear regression models explaining the average leaf soluble carbohydrates, free amino acids, starch, total phenolics, carbon and nitrogen contents and the C:N ratio of different species.

Life form	Genus	Dependent variable	*df*	*P*	*r*^2^_adj_	Linear model
Submerged macrophytes	*P. pectinatus*	Ln C	(1, 22)	0.003	0.310	5.721***+ 0.024** ln altitude
	*P. perfoliatus*	Ln C	(1, 4)	0.009	0.814	7.675*** – 0.534** ln latitude
		Ln C:N	(1, 4)	0.017	0.747	−0.339 + 1.173* ln MAT+5
			(2, 3)	0.006	0.947	1.614 – 0.257* ln altitude + 1.170** ln MAT+5
	*P. malaianus*	Ln TOPH	(1, 20)	0.012	0.240	8.207** – 1.891* ln latitude
		Ln C	(1, 16)	0.003	0.398	7.737*** – 0.548** ln latitude
	*P. maackianus*	Ln starch	(1, 14)	0.006	0.380	−7.315*+ 3.489** ln MAT+5
			(2, 13)	0.003	0.543	−20.374**+ 4.300* ln latitude + 3.039** ln MAT+5
			(3, 12)	0.001	0.670	−63.164**+ 14.426** ln latitude + 0.399* ln altitude + 5.392** ln MAT+5
		Ln N	(1, 11)	0.006	0.463	6.708*** – 1.212** ln Altitude
		Ln C:N	(1, 11)	0.008	0.446	−0.253 + 1.010** ln MAT+5
	*P. crispus*	Ln TOPH	(1, 21)	¡0.001	0.491	1.143***+ 0.165*** ln Altitude
		Ln N	(1, 20)	0.003	0.324	−2.103 + 1.779** ln MAT+5
		Ln C:N	(1, 20)	0.005	0.304	7.639*** – 1.687** ln MAT+5
			(2, 19)	0.002	0.421	7.924***+ 0.051* ln altitude – 1.835** ln MAT+5
	*N. minor*	Ln C	(1, 3)	0.002	0.958	12.060*** – 1.838** ln latitude
		Ln N	(1, 3)	¡0.001	0.996	10.643*** – 2.186*** ln latitude
	*C. demersum*	Ln N	(1, 25)	0.010	0.206	−2.356 + 1.681* ln latitude
		Ln C:N	(1, 25)	0.002	0.287	8.478*** – 1.780** ln latitude
	*M. verticillatum*	Ln TOPH	(1, 42)	0.001	0.230	13.481*** – 3.267** ln latitude
		Ln C	(1, 35)	0.001	0.271	8.345*** – 0.749** ln latitude
		Ln N	(1, 35)	0.007	0.167	1.840***+ 0.408** ln MAT+5
		Ln C:N	(1, 35)	0.002	0.212	3.956*** - 0.415** ln MAT+5
	*V. natans*	Ln C:N	(1, 23)	0.003	0.305	6.542*** – 1.196** ln latitude
Floating-leaved macrophytes	*T. bispinosa*	Ln FAA	(1, 35)	0.002	0.227	10.493** – 3.710** ln MAT+5
		Ln C	(1, 35)	0.005	0.180	5.895***+ 0.017** ln altitude
	*H. dubia*	Ln C	(1, 13)	0.017	0.314	6.828*** – 0.269* ln latitude
	*N. peltatum*	Ln N	(1, 9)	0.017	0.428	9.085 – 1.640* ln Latitude
Emergent macrophytes	*N. nucifera*	Ln TOPH	(1, 7)	0.001	0.787	11.411*** – 2.367** ln latitude
	*S. validus*	Ln C	(1, 4)	0.016	0.751	5.829***+ 0.054* ln MAT+5
	*T. orientalis*	Ln SC	(1, 11)	0.004	0.497	3.266***+ 0.117** ln altitude
		Ln TOPH	(1, 11)	0.010	0.423	2.455***+ 0.102* ln altitude
		Ln N	(1, 10)	0.011	0.439	3.277*** – 0.132* ln altitude
		Ln C:N	(1, 10)	0.007	0.481	2.718***+ 0.134** ln altitude
Free-floating macrophytes	*L. minor*	Ln C:N	(1, 5)	0.005	0.786	0.626 + 0.575** ln MAT+5
		Ln TOPH	(1, 5)	0.001	0.872	−4.274**+ 1.921** ln latitude

The models result from a forward stepwise selection procedure using the independent variables latitude, altitude and mean annual temperature. All significant (*P* < 0.05/3) regression models are shown. All dates were log-transformed (base *e*), the significance of the regression cofficients is indicated by *P < 0.05, **P < 0.01, ***P < 0.001, without asterisk *P* > 0.05. Bonferroni correction significance level threshold value, *P* = 0.05/3.

## Discussion

We found that the C and N stoichiometry of 24 aquatic macrophytes varied greatly across geographic and climatic gradients in China, indicating physiological plasticity. Considering all species, the C and N contents decreased significantly from low to high latitudes, which is in agreement with the results in the study of 753 terrestrial plants from across China undertaken by Han et al. (2005) and of 122 aquatic macrophytes in the eastern part of China (Xia et al., 2014), though the variance explained by the linear regression models in our study was low. In China, climatic factors and human activities vary greatly across the country’s large geographic scales; thus, southern (lower latitude) areas experience higher temperatures and precipitation, less cloudiness and frost and more severe eutrophication than northern areas (Wu et al., 1999). This benefits the growth and assimilation of inorganic carbon and nutrients of aquatic plants, which might have contributed to the finding of enhanced C and N contents. In the present study, the C content of several aquatic species tended to increase with increasing altitude (though low variance was explained, which may be due to lack of nutrient data), possibly reflecting the fact that plants require higher C storage at the low CO_2_ pressure in the Tibetan plateau (He et al., 2006; Yang et al., 2014). Although significant temperature changes occur across the large geographic scale applied in our study, it is surprising that the C and N contents did not correlate with MAT, implying that physiological processes might depend more on C and N availability (i.e., stem openness and surface area of roots, Cramer et al., 2001) than on MAT-related adjustment of enzyme activity (Reich and Oleksyn, 2004). SC, FAA, starch, and TOPH contents did not correlate with latitude, altitude and MAT. SC, FAA, starch, and TOPH are intermediates of C and N metabolim and account for a small proportion of the C and N contents, and they may therefore respond rapidly and flexibly to local habitat alterations. This contrasts with the contents of C and N that are largely structural compounds in plant tissue and may be indicative of changes in the regional environment (Elser et al., 2000; Reich and Oleksyn, 2004; Han et al., 2005, 2011; He et al., 2006).

We found significant differences in the contents of SC, FAA, starch, TOPH, C, and N and in C:N ratios among the macrophytes with different life forms. As CO_2_ diffuses 10,000 times less in water than in air and light availability attenuates exponentially through the water column, inorganic C and light availability vary extensively from the shore to the deep water (Cao et al., 2008). Expectedly, submerged macrophytes experience much lower CO_2_ and light availability than emergent and free-floating macrophytes, while floating-leaved macrophytes experience intermediate CO_2_ and light availability in fluctuating water (Cao et al., 2008), and these factors may contribute to the differences in C contents. Aquatic macrophytes can take up nutrients from both sediment and water (Rattray et al., 1991; Madsen and Cedergreen, 2010; Cao et al., 2011; Li et al., 2013). Rooted macrophytes absorb nutrients mainly from the sediment, while free-floating macrophytes primarily absorb nutrients from the water column (Barko and Smart, 1986; Fernández-Aláez et al., 1999). We found that the N content was significantly higher in the free-floating plants than in the other life forms, which is in line with the results reported by Xia et al. (2014), possibly reflecting the simplified mechanical support structure required by this life form compared with the other forms studied (Bonser and Geber, 2005; Weijschedé et al., 2006).

The C metabolic pathway of all aquatic macrophytes in the current study were C3. At life forms level, a significant correlation was found between stoichiometric characteristics such as C, N or C:N with one or more climatic variables (latitude, altitude, and MAT) for submerged macrophytes (*P. pectinatus*, *P. perfoliatus*, *P. malaianus*, *P. maackianus*, *P. crispus*, *N. minor*, *C. demersum*, *M. verticillatum*, and *V. natans*), while the other life forms characteristics were not better related to the climate variables. Whether this difference reflects differences in the access to C (lower for submerged macrophytes) and N with implications for the metabolites remains to be elucidated. However, the correlations of leaf stoichiometric characteristics with the three climate variables were more significant for generalist species (e.g., *P. maackianus*, *P. crispus*, and *M. verticillatum*), than for specialized species. This indicates a more obvious physiological plasticity of generalist species across the geographic and climatic gradients in China, which is useful information for lake managers in the restoration of lost aquatic plants in lakes different climate regions.

Our study allows us to draw the following conclusions: (1) the C and N stoichiometry of aquatic macrophytes varied greatly across the large geographic and climatic study gradient; (2) among the different life forms, the C and N contents related more closely than the C and N metabolic indicators to latitude, altitude, or MAT for the submerged macrophyte life form, while the relationships with metabolic indicators turned out to be insignificant for most species of the other life forms. As we have no nutrient data from these sites we cannot fully rule out that some of the relationships we found are not affected, in part, by systematic variation in nutrient concentrations along the climate gradients used (latitude, altitude, and MAT).

## Author Contributions

QC was responsible for sample processing, data analysis, and draft completion. TC was responsible for the collection of samples. LN was responsible for samples collection. PX gaves guidance during sample processing and data analysis. EJ gave his opinions during the data analysis process and gave a lot of comments during the revision of the draft.

## Conflict of Interest Statement

The authors declare that the research was conducted in the absence of any commercial or financial relationships that could be construed as a potential conflict of interest. The handling Editor is currently co-organizing a Research Topic with one of the authors TC, and confirms the absence of any other collaboration.

## Footnotes

1 https://www.wunderground.com/
