# Supplementary material for: Leaf Soluble Carbohydrates, Free Amino Acids, Starch, Total Phenolics, Carbon and Nitrogen Stoichiometry of 24 Aquatic Macrophyte Species Along Climate Gradients in China
Source: Front Plant Sci. 2019 Apr 11;10:442. doi: 10.3389/fpls.2019.00442 (PMC6470362; doi:10.3389/fpls.2019.00442)
Supplement: Supplementary file 1 [file Table_1.docx]

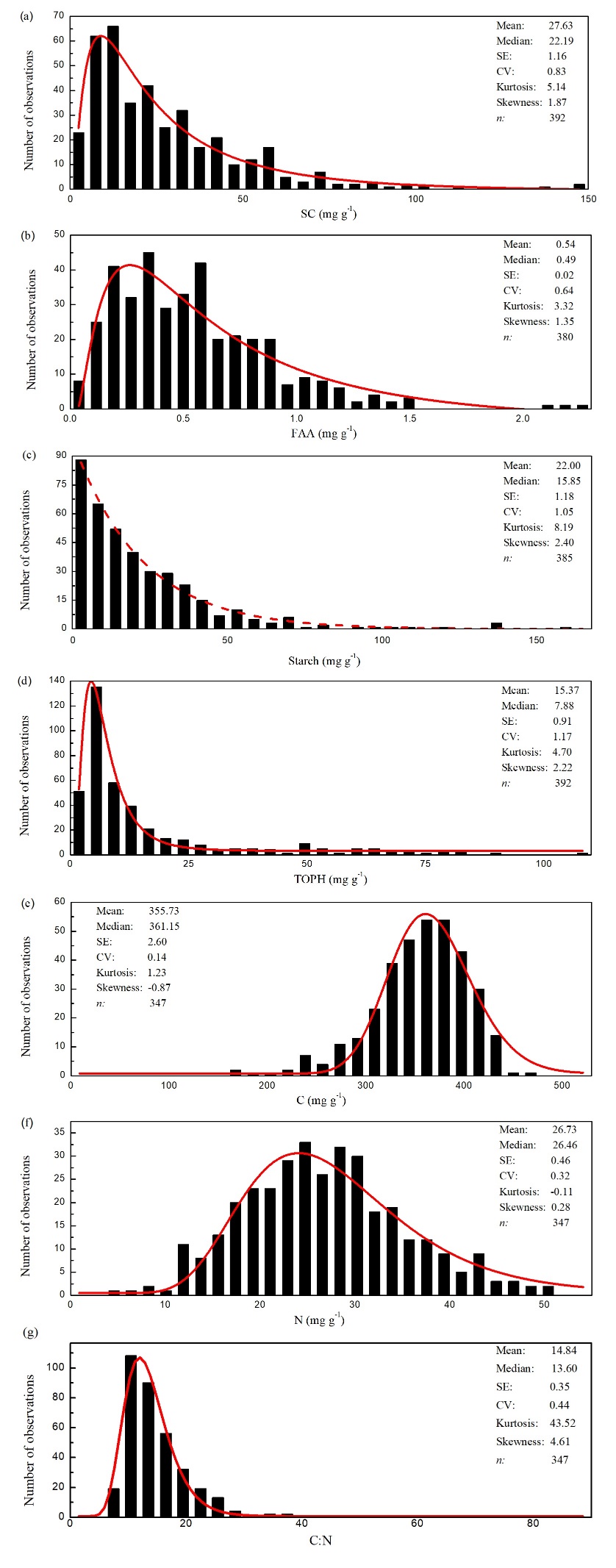


**Fig S1** Histograms showing the distribution of leaf soluble carbohydrates (mg g^-1^) (a); free amino acids (mg g^-1^) (b); starch (mg g^-1^) (c); total phenolics (mg g^-1^) (d); carbon (mg g^-1^) (e); nitrogen (mg g^-1^) (f) and the C:N ratio (g). Red solid curves in (a-g) indicate fitted log-normal curves; red dashed curves in (c) indicate fitted exponential curves. The data include all the species considered in the study.

**Table S1** Arithmetic mean and standard error of leaf soluble carbohydrates, free amino acids, starch, total phenolics, carbon and nitrogen contents and the C:N ratio of aquatic macrophytes with different life forms.

| Life form | SC (mg g^-1^) | |  | FAA(mg g^-1^) | |  | Starch (mg g^-1^) | |  | TOPH (mg g^-1^) | |  | C (mg g^-1^) | |  | N (mg g^-1^) | |  | C:N | |
| --- | --- | --- | --- | --- | --- | --- | --- | --- | --- | --- | --- | --- | --- | --- | --- | --- | --- | --- | --- | --- |
|  | *n* | Mean±SE |  | *n* | Mean±SE |  | *n* | Mean±SE |  | *n* | Mean±SE |  | *n* | Mean±SE |  | *n* | Mean±SE |  | *n* | Mean±SE |
| Submerged macrophytes |  |  |  |  |  |  |  |  |  |  |  |  |  |  |  |  |  |  |  |  |
| *P. pectinatus* | 27 | 26.42±3.74 |  | 26 | 0.54±0.07 |  | 27 | 17.65±4.08 |  | 27 | 5.80±0.56 |  | 24 | 344.63±7.19 |  | 24 | 21.26±1.63 |  | 24 | 18.14±1.18 |
| *P. perfoliatus* | 7 | 39.08±5.63 |  | 7 | 0.45±0.10 |  | 7 | 25.73±8.10 |  | 7 | 8.59±1.32 |  | 6 | 359.46±16.16 |  | 6 | 20.86±3.60 |  | 6 | 19.09±2.38 |
| *P. lucens* | 5 | 32.10±10.95 |  | 5 | 0.44±0.04 |  | 5 | 9.47±2.70 |  | 5 | 5.80±1.30 |  | 4 | 370.29±13.32 |  | 4 | 21.54±2.69 |  | 4 | 17.74±1.51 |
| *P. malaianus* | 24 | 26.82±3.26 |  | 24 | 0.49±0.05 |  | 23 | 28.58±5.91 |  | 24 | 7.55±0.94 |  | 19 | 353.76±7.86 |  | 19 | 24.47±1.44 |  | 19 | 15.33±0.88 |
| *P. maackianus* | 17 | 19.24±2.23 |  | 17 | 0.37±0.06 |  | 16 | 31.06±7.62 |  | 17 | 5.07±0.37 |  | 13 | 351.86±11.48 |  | 13 | 23.92±2.57 |  | 13 | 15.99±1.12 |
| *N. minor* | 6 | 41.08±21.35 |  | 5 | 0.39±0.14 |  | 6 | 27.24±10.41 |  | 6 | 9.92±2.25 |  | 5 | 310.30±37.33 |  | 5 | 22.85±3.18 |  | 5 | 13.80±0.47 |
| *N. marina* | 6 | 33.05±10.84 |  | 4 | 0.66±0.16 |  | 6 | 9.21±2.30 |  | 6 | 20.69±13.54 |  | 4 | 320.17±13.38 |  | 4 | 30.52±4.48 |  | 4 | 11.13±1.57 |
| *H. verticillata* | 22 | 17.99±3.26 |  | 22 | 0.51±0.07 |  | 22 | 21.56±4.02 |  | 22 | 5.20±0.54 |  | 18 | 324.27±8.83 |  | 18 | 27.78±1.63 |  | 18 | 12.28±0.73 |
| *C. demersum* | 31 | 12.77±1.03 |  | 30 | 0.69±0.09 |  | 31 | 20.95±5.42 |  | 31 | 6.21±0.62 |  | 28 | 330.81±9.01 |  | 28 | 31.15±1.67 |  | 28 | 11.59±0.85 |
| *M. verticillatum* | 45 | 18.18±1.78 |  | 44 | 0.54±0.05 |  | 44 | 35.10±4.48 |  | 45 | 14.06±1.64 |  | 38 | 323.32±7.55 |  | 38 | 21.85±1.10 |  | 38 | 16.53±1.03 |
| *V. natans* | 27 | 11.30±1.17 |  | 26 | 0.43±0.07 |  | 26 | 22.81±3.18 |  | 27 | 4.91±0.49 |  | 24 | 312.91±8.76 |  | 24 | 27.23±1.15 |  | 24 | 11.80±0.47 |
| *P. crispus* | 24 | 23.72±4.21 |  | 24 | 0.47±0.06 |  | 23 | 21.82±3.26 |  | 24 | 6.14±0.64 |  | 23 | 338.74±9.10 |  | 23 | 26.90±1.36 |  | 23 | 13.26±0.75 |
| Floating-leaved macrophytes |  |  |  |  |  |  |  |  |  |  |  |  |  |  |  |  |  |  |  |  |
| *P . natans* | 8 | 32.19±7.33 |  | 8 | 0.40±0.09 |  | 8 | 18.63±5.82 |  | 8 | 11.27±2.52 |  | 5 | 381.88±28.37 |  | 5 | 29.54±5.94 |  | 5 | 14.97±2.61 |
| *T. bispinosa* | 38 | 35.24±2.84 |  | 38 | 0.58±0.05 |  | 37 | 20.37±2.87 |  | 38 | 48.65±3.46 |  | 38 | 377.96±5.82 |  | 38 | 27.66±1.15 |  | 38 | 14.47±0.62 |
| *E. ferox* | 4 | 39.75±12.72 |  | 3 | 0.43±0.13 |  | 4 | 29.92±17.64 |  | 4 | 62.99±9.71 |  | 4 | 387.72±13.53 |  | 4 | 26.07±2.00 |  | 4 | 15.26±1.70 |
| *H. dubia* | 18 | 25.15±5.27 |  | 17 | 0.70±0.07 |  | 18 | 28.66±4.64 |  | 18 | 9.22±1.40 |  | 16 | 364.29±5.13 |  | 16 | 32.23±2.00 |  | 16 | 12.07±0.87 |
| *N. peltatum* | 11 | 59.70±8.34 |  | 10 | 0.56±0.08 |  | 10 | 13.10±2.31 |  | 11 | 16.10±1.79 |  | 11 | 398.42±8.05 |  | 11 | 31.57±2.75 |  | 11 | 13.71±1.31 |
| *P. amphibium* | 11 | 27.59±5.15 |  | 11 | 0.64±0.08 |  | 11 | 28.58±4.80 |  | 11 | 55.11±5.18 |  | 11 | 423.49±4.52 |  | 11 | 31.34±2.08 |  | 11 | 14.03±0.81 |
| Emergent macrophytes |  |  |  |  |  |  |  |  |  |  |  |  |  |  |  |  |  |  |  |  |
| *Z. latifolia* | 16 | 50.95±5.80 |  | 15 | 0.40±0.06 |  | 16 | 5.63±2.39 |  | 16 | 13.27±1.90 |  | 14 | 402.93±3.85 |  | 14 | 26.84±1.42 |  | 14 | 15.67±0.98 |
| *N. nucifera* | 9 | 39.48±7.20 |  | 9 | 0.52±0.09 |  | 9 | 6.35±2.11 |  | 9 | 31.50±4.38 |  | 8 | 404.32±12.73 |  | 8 | 35.48±3.37 |  | 8 | 12.05±1.08 |
| *S. validus* | 7 | 91.10±16.55 |  | 7 | 0.44±0.12 |  | 7 | 6.30±3.29 |  | 7 | 14.87±1.96 |  | 7 | 396.44±2.51 |  | 7 | 20.12±4.02 |  | 7 | 29.16±9.86 |
| *T. orientalis* | 13 | 41.53±4.14 |  | 12 | 0.42±0.07 |  | 13 | 2.77±0.73 |  | 13 | 17.34±1.45 |  | 12 | 449.4±6.42 |  | 12 | 18.45±1.78 |  | 12 | 24.24±2.25 |
| Free-floating macrophytes |  |  |  |  |  |  |  |  |  |  |  |  |  |  |  |  |  |  |  |  |
| *E. crassipes* | 9 | 20.77±5.75 |  | 9 | 0.90±0.20 |  | 9 | 12.60±3.17 |  | 9 | 9.59±2.09 |  | 8 | 380.67±10.73 |  | 8 | 37.15±3.31 |  | 8 | 10.84±1.00 |
| *L. minor* | 7 | 11.72±3.62 |  | 7 | 0.81±0.26 |  | 7 | 43.60±16.20 |  | 7 | 7.88±0.68 |  | 7 | 352.59±13.40 |  | 7 | 33.79±1.71 |  | 7 | 10.53±0.39 |

**Table S2** The coefficient of variation between (intraspecific) and within ( interspecies) aquatic macrophytes with different life forms relative to leaf soluble carbohydrates, free amino acids, starch, total phenolics, carbon and nitrogen contents and the C:N ratio.

| Life form | *CV* | | | | | | |
| --- | --- | --- | --- | --- | --- | --- | --- |
|  | SC | FAA | Starch | TOPH | C | N | C:N |
| Submerged macrophytes | 0.39 | 0.20 | 0.35 | 0.56 | 0.06 | 0.14 | 0.19 |
| *P. pectinatus* | 0.74 | 0.62 | 1.20 | 0.50 | 0.10 | 0.37 | 0.32 |
| *P. perfoliatus* | 0.38 | 0.57 | 0.83 | 0.41 | 0.11 | 0.42 | 0.31 |
| *P. lucens* | 0.76 | 0.18 | 0.64 | 0.50 | 0.07 | 0.25 | 0.17 |
| *P. malaianus* | 0.60 | 0.48 | 0.99 | 0.61 | 0.10 | 0.26 | 0.25 |
| *P. maackianus* | 0.48 | 0.63 | 0.98 | 0.30 | 0.12 | 0.39 | 0.25 |
| *N. minor* | 1.27 | 0.78 | 0.94 | 0.56 | 0.27 | 0.31 | 0.08 |
| *N. marina* | 0.80 | 0.49 | 0.61 | 1.60 | 0.08 | 0.29 | 0.28 |
| *H. verticillata* | 0.85 | 0.66 | 0.87 | 0.49 | 0.12 | 0.25 | 0.25 |
| *C. demersum* | 0.45 | 0.71 | 1.44 | 0.56 | 0.14 | 0.28 | 0.39 |
| *M. verticillatum* | 0.66 | 0.61 | 0.85 | 0.78 | 0.14 | 0.31 | 0.38 |
| *V. natans* | 0.54 | 0.81 | 0.71 | 0.52 | 0.14 | 0.21 | 0.20 |
| *P. crispus* | 0.87 | 0.63 | 0.72 | 0.51 | 0.13 | 0.24 | 0.27 |
| Floating-leaved macrophytes | 0.34 | 0.21 | 0.30 | 0.72 | 0.05 | 0.08 | 0.08 |
| *P . natans* | 0.64 | 0.67 | 0.88 | 0.63 | 0.17 | 0.45 | 0.39 |
| *T. bispinosa.* | 0.50 | 0.55 | 0.86 | 0.44 | 0.09 | 0.26 | 0.26 |
| *E. ferox* | 0.64 | 0.52 | 1.18 | 0.31 | 0.07 | 0.15 | 0.22 |
| *H. dubia* | 0.89 | 0.39 | 0.69 | 0.64 | 0.06 | 0.25 | 0.29 |
| *N. peltatum* | 0.46 | 0.46 | 0.56 | 0.37 | 0.07 | 0.29 | 0.32 |
| *P. amphibium* | 0.62 | 0.39 | 0.56 | 0.31 | 0.04 | 0.22 | 0.19 |
| Emergent macrophytes | 0.43 | 0.12 | 0.32 | 0.43 | 0.01 | 0.31 | 0.39 |
| *Z. latifolia* | 0.46 | 0.57 | 1.70 | 0.57 | 0.04 | 0.20 | 0.23 |
| *N. nucifera* | 0.55 | 0.54 | 1.00 | 0.42 | 0.09 | 0.27 | 0.25 |
| *S. validus* | 0.48 | 0.69 | 1.38 | 0.35 | 0.02 | 0.53 | 0.89 |
| *T. orientalis* | 0.36 | 0.61 | 0.95 | 0.30 | 0.05 | 0.33 | 0.32 |
| Free-floating macrophytes | 0.39 | 0.08 | 0.78 | 0.14 | 0.05 | 0.07 | 0.02 |
| *E. crassipes* | 0.83 | 0.68 | 0.75 | 0.65 | 0.08 | 0.25 | 0.26 |
| *L. minor* | 0.82 | 0.86 | 0.98 | 0.23 | 0.10 | 0.13 | 0.10 |
| **Different life form** | 0.46 | 0.25 | 0.51 | 0.50 | 0.08 | 0.15 | 0.27 |
| **All species** | 0.54 | 0.26 | 0.52 | 0.99 | 0.09 | 0.19 | 0.29 |

**Table S3** Pearson correlation among leaf soluble carbohydrates, free amino acids, starch, total phenolics, carbon and nitrogen contents and the C:N ratio of aquatic macrophytes and latitude, mean annual temperature and altitude in China.

|  | Latitude | Altitude | MAT | SC | FAA | Starch | TOPH | C | N | C:N |
| --- | --- | --- | --- | --- | --- | --- | --- | --- | --- | --- |
| Latitude | 1.00 | -0.59^***^ | -0.23^***^ | 0.10^*^ | 0.02 | -0.01 | -0.09 | -0.29^***^ | -0.17^**^ | 0.06 |
| Altitude |  | 1.00 | -0.33^***^ | 0.01 | -0.07 | 0.02 | 0.05 | 0.25^***^ | -0.07 | 0.19^**^ |
| MAT |  |  | 1.00 | -0.12^*^ | -0.01 | 0.11^*^ | -0.02 | 0.02 | 0.05 | -0.04 |
| SC |  |  |  | 1.00 | 0.03 | -0.02 | 0.47^***^ | 0.37^***^ | -0.23^***^ | 0.40^***^ |
| FAA |  |  |  |  | 1.00 | -0.17^**^ | 0.12^*^ | 0.07 | 0.38^***^ | -0.36^***^ |
| Starch |  |  |  |  |  | 1.00 | 0.02 | 0.01 | -0.15^**^ | 0.16^**^ |
| TOPH |  |  |  |  |  |  | 1.00 | 0.59^***^ | 0.17^**^ | 0.09 |
| C |  |  |  |  |  |  |  | 1.00 | 0.30^***^ | 0.14^*^ |
| N |  |  |  |  |  |  |  |  | 1.00 | -0.91^***^ |
| C:N |  |  |  |  |  |  |  |  |  | 1.00 |

All dates were log-transformed (base *e*), * *P*<0.05, ** *P*<0.01, *** *P*<0.001, without asterisk *P*>0.05.

**Table S4** Pearson correlation among leaf soluble carbohydrates, free amino acids, starch, total phenolics, carbon and nitrogen contents and the C:N ratio of different life forms and latitudes, mean annual temperatures and altitudes in China.

| Life form |  | index |  | North Latitude |  | MAT |  | Altitude |
| --- | --- | --- | --- | --- | --- | --- | --- | --- |
| Submerged macrophytes |  | SC |  | 0.06 |  | -0.15* |  | 0.11 |
|  |  | FAA |  | 0.06 |  | -0.05 |  | -0.10 |
|  |  | Starch |  | -0.09 |  | 0.12 |  | 0.01 |
|  |  | TOPH |  | -0.24*** |  | -0.12 |  | 0.19** |
|  |  | C |  | -0.39*** |  | -0.02 |  | 0.32*** |
|  |  | N |  | -0.12 |  | 0.13 |  | -0.20** |
|  |  | C:N |  | -0.06 |  | -0.15* |  | 0.35*** |
| Floating-leaved macrophytes |  | SC |  | 0.14 |  | 0.17 |  | -0.10 |
|  |  | FAA |  | 0.02 |  | -0.15 |  | -0.14 |
|  |  | Starch |  | 0.09 |  | 0.29* |  | -0.06 |
|  |  | TOPH |  | -0.03 |  | 0.27* |  | -0.14 |
|  |  | C |  | -0.27* |  | 0.52*** |  | 0.26* |
|  |  | N |  | -0.18 |  | -0.04 |  | 0.11 |
|  |  | C:N |  | 0.11 |  | 0.23* |  | 0.04 |
| Emergent macrophytes |  | SC |  | 0.01 |  | -0.29 |  | 0.22 |
|  |  | FAA |  | -0.14 |  | -0.09 |  | 0.24 |
|  |  | Starch |  | 0.33* |  | -0.13 |  | -0.06 |
|  |  | TOPH |  | -0.15 |  | 0.17 |  | 0.22 |
|  |  | C |  | -0.16 |  | 0.17 |  | 0.12 |
|  |  | N |  | -0.19 |  | 0.01 |  | -0.16 |
|  |  | C:N |  | 0.18 |  | 0.01 |  | 0.18 |
| Free-floating macrophytes |  | SC |  | 0.24 |  | -0.05 |  | -0.14 |
|  |  | FAA |  | 0.15 |  | -0.03 |  | -0.29 |
|  |  | Starch |  | 0.15 |  | -0.29 |  | 0.12 |
|  |  | TOPH |  | 0.51** |  | -0.36 |  | -0.02 |
|  |  | C |  | 0.34 |  | -0.27 |  | 0.11 |
|  |  | N |  | -0.06 |  | -0.30 |  | 0.18 |
|  |  | C:N |  | 0.21 |  | 0.15 |  | -0.11 |

All dates were log-transformed (base *e*), * *P*<0.05, ** *P*<0.01, *** *P*<0.001, without asterisk *P*>0.05.
